# Periodontal infection as a risk factor for preterm low birth weight

**DOI:** 10.4103/0972-124X.70832

**Published:** 2010

**Authors:** D. Gandhimadhi, R. Mythili

**Affiliations:** *Department of Periodontics, Mahatma Gandhi Postgraduate Institute of Dental Sciences, Indira Nagar, Puducherry, Tamil Nadu, India*; 1 *Rajah Muthiah Dental College and Hospital, Annamalai University, Chidambaram, Tamil Nadu, India*

**Keywords:** Periodontal medicine, preterm low birth weight

## Abstract

**Introduction:**

There is an overwhelming body of evidence strongly suggesting that periodontal infection may have a significant negative impact on pregnancy outcome in some women. The aim of this study was to determine the association, if any, between periodontal disease and preterm low birth weight.

**Materials and Methods:**

A total of 211 mothers between the ages of 17 and 35 were grouped into two categories based on the gestational age and weight of the baby as cases (< 37 weeks, < 2500 g) and controls (>37 weeks, >2500 g). Relevant obstetric history and information on other primary risk factors for preterm low birth weight were obtained. Investigation reports on blood group, Rh factor and hemoglobin (Hb) were also gathered. Oral assessments included: simplified oral hygiene index (OHI-S), gingival bleeding index, probing pocket depth and clinical attachment level (CAL).

**Results:**

Cases had significantly more attachment loss and probing pocket depth, poor oral hygiene, more percentage of sites with attachment loss (Extent) and more mean attachment loss per site (Severity) and less Hb than controls. The number of visits for prenatal care and the percentage of sites with CAL≥2mm (Extent 2) remained significant when compared to other variables.

**Conclusions:**

The study indicated that periodontal disease is a contributing factor for preterm low birth weight.

## INTRODUCTION

Preterm birth is a major medical, social and economic problem, accounting for a large proportion of maternal and especially neonatal mortality, acute morbidity and long-term sequelae.[1] The normal gestation for humans, full term, is 40 weeks. Preterm or premature birth is usually defined as a gestational age of less than 37 weeks. The majority of preterm births are also low birth weight [LBW- less than 2500 g (upto and including 2499 g)].[2]

Among the risk factors for preterm low birth weight (PTLBW) deliveries are high or low maternal age, inadequate prenatal care, smoking, tobacco use, alcohol or drug use during pregnancy, genetic background, low socioeconomic status, maternal stress and genitourinary tract infections. However, these risk factors are not present in approximately one fourth of preterm low birth weight deliveries, leading to a continued search for other causes.[3,4]

There is increasing evidence that infection may play a role in preterm delivery. Numerous studies have reported significantly increased risks for preterm labor, premature rupture of membranes and preterm delivery among women with genitourinary tract infection,[4] vaginal colonization of black pigmented *Bacteroides*,[3] positive amniotic fluid cultures of *Fusobacterium*[1] and histologic chorioamnionitis of the extraplacental membranes.[4]

Throughout gestation, amniotic levels of prostaglandins, especially prostaglandin E_2_ (PGE_2_), rise steadily until a sufficient level is reached to induce normal labor and delivery.[3]

Maternal genitourinary infection may lead to the presence of amnionic bacterial products such as lipopolysaccharides (LPS), or endotoxin, from gram-negative organisms, which stimulate production of host-derived (maternal/ fetal) cytokines in the amnion and decidua. These cytokines, including interleukin-1 (IL-1), tumor necrosis factor-alpha (TNF-α) and interleukin-6 (IL-6), stimulate increased prostaglandin production from the amnion and decidua, leading to the onset of preterm labor.[3]

The premature increase in PGE_2_ and PGF_2α_ is characteristic of preterm labor whether or not clinical or subclinical maternal genitourinary infection is detected. The question then arises as to what stimulates the increased cytokine levels and the resultant increased prostaglandin levels seen in preterm delivery in patients with no evidence of genitourinary tract infection. This observation supports the current opinion that many cases of PTLBW are a result of infections of unknown origin, that is, infections originating in areas other than the genitourinary tract.[3]

In periodontal disease when periodontal pockets form, the pocket epithelium is the only barrier between the biofilms and connective tissue. The strands of thin frequently ulcerated epithelium are easily broached, allowing bacterial access to the connective tissue and blood vessels. In patients with moderate-to-severe periodontitis, the total area of the pocket epithelium in direct contact with the subgingival biofilms is surprisingly large (72 cm^2^); it may be about the size of the palm of the human hand, and in case of advanced disease it is much larger.

There is evidence that significant doses of viable gramnegative bacteria, LPS and other soluble bacterial components have ready access to the connective tissue and enter the circulation.[5]

Based on all of the above observations, it has been hypothesized that oral infection presented by the gramnegative anaerobic bacterial challenge can serve as a chronic reservoir for hematogenous translocation of bacteria or bacterial products such as LPS to the fetoplacental unit. It is also possible that cytokines, such as TNF-α, which are produced by the infected periodontium and appear in the systemic circulation, can target the placenta. However, it seems more likely that blood-borne bacteria and/or bacterial products, especially LPS, target the placenta to mediate local PGE_2_ and TNF-α synthesis.[6]

The clear role of subject-based and environmental factors in the clinical outcome of the interplay between periodontal bacteria and the host response emphasizes the intimate link between oral and systemic health. This in turn has led to an examination of the hypothesis that persistent, gram-negative bacterial challenge and concomitant unregulated or deregulated host response associated with periodontal diseases may have consequences that extend beyond the periodontal tissues themselves.[7]

The present study was aimed to determine the association, if any, between periodontal disease and preterm low birth weight by measuring the clinical parameters of periodontal disease status in patients who delivered a low birth weight (<2500 g) baby before term (<37 weeks) and comparing them with patients who delivered a normal birth weight (>2500 g) baby at term (>37 weeks).

## MATERIALS AND METHODS

Patients were selected depending on the gestational age and weight of the baby. A total of 211 mothers with age ranging from 17 to 35 years were examined, and a case-control study of this sample was performed. All mothers were examined within 5 days postpartum.

The study was conducted at the Government Maternity Hospital, Pondicherry.

### Inclusion criteria

Patients with preterm labor [preterm labor (PTL) is defined as contractions and cervical changes necessitating medical intervention] or premature rupture of membranes [premature rupture of membranes (PROM) — rupture of membranes (extraplacental chorioamnionic) occurring before the onset of labor and not resulting in labor within 6 hours of rupture] and gestational age less than 37 weeks with birth weight of the baby being less than 2.5 kg were included in the case category {109 mothers}.

Patients with normal labor and gestational age more than 37 weeks with birth weight of the baby being more than 2.5 kg were included in the control category {102 mothers}.

### Exclusion criteria

Mothers who had a history of cardiovascular problems, eclampsia, respiratory disorders and those who had undergone cesarean section.

### The following parameters were recorded

Obstetric factors such as maternal age at delivery, number of pregnancies, previous induced abortions and spontaneous abortions, previous preterm deliveries, previous full-term deliveries and live births, respectively, were recorded. Onset of prenatal care — whether prior to 20 weeks, between 20 and 25 weeks, after 25 weeks or no prenatal care and the total number of prenatal visits, use of tobacco and alcohol were also noted. Other outcome variables, like hypertension, genitourinary tract infection, were recorded, and investigation reports on blood group, Rh factor and hemoglobin were also gathered. Oral examination was carried out using artificial light, a mouth mirror, explorer and William’s periodontal probe. The clinical parameters recorded were ‘simplified oral hygiene index,’ gingival bleeding index (Ainamo and Bay), probing pocket depth and clinical attachment level.

After recording all the clinical parameters, the Extent and Severity of attachment loss were derived from the clinical attachment level.

Extent of attachment loss[8] - Percentage of sites with CAL≥2, 3 or 4 mm, which is Extent 2 (E2), Extent 3 (E3) or Extent 4 (E4), respectively.

It is calculated using the formula[9]

$$E\;=\;\left(\sum_{i=1}^{n}d_{i}\;\times \;100\right)/n$$

Severity of attachment loss[8] - Mean CAL in sites where CAL≥2, 3 or 4 mm, which is Severity 2 (S2), Severity 3 (S3) or Severity 4 (S4), respectively.

It is calculated using the formula[9]

$$S\;=\;\sum_{i=1}^{n}\left[d_{i}\;\left(x_{i}-1\right)\right]/\sum d_{i,}$$

where n=number of sites actually examined,

x_i_= measurement of attachment level,

x= runs from 1 to n, i.e., x_i_=(x_1_, x_2_, x_3_ …… x_n_),

∑= summation, and

d_i_=attachment loss exceeding 1 mm, i.e., d_i_=1 if x_i_>1 and d_i_=0 otherwise.

### Statistical analysis

The statistical package SPSS PC+ (Statistical Package for Social Science, version 4.01) was used for statistical analysis. Mean±standard deviation and proportion were estimated from the sample for each study group.

The mean values were compared by Student independent *t* test. Proportions were compared by chi-square test/ Fisher exact test appropriately. Multiple logistic regression analysis was done to identify the predictive factors to the ‘cases.’

In the present study, *P*<.05 was considered as the level of significance.

## RESULTS

Analysis of the data obtained from the 211 mothers who were examined gave the following results.

### Inference

**Figure 1:**
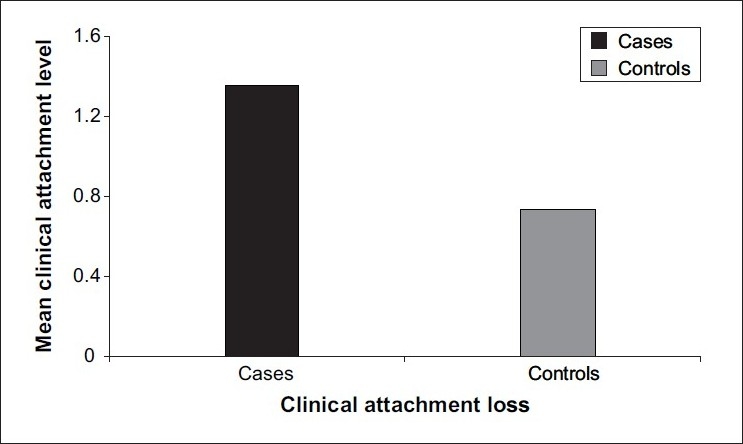
Mean value of clinical attachment loss in cases and controls

**Figure 2:**
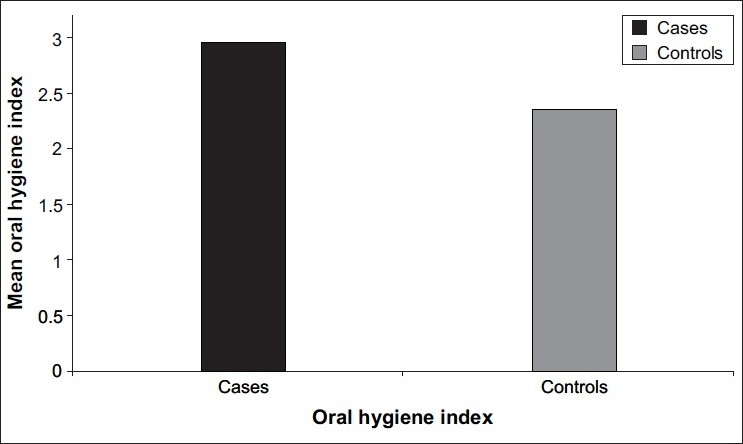
Mean value of oral hygiene index in cases and controls

**Figure 3:**
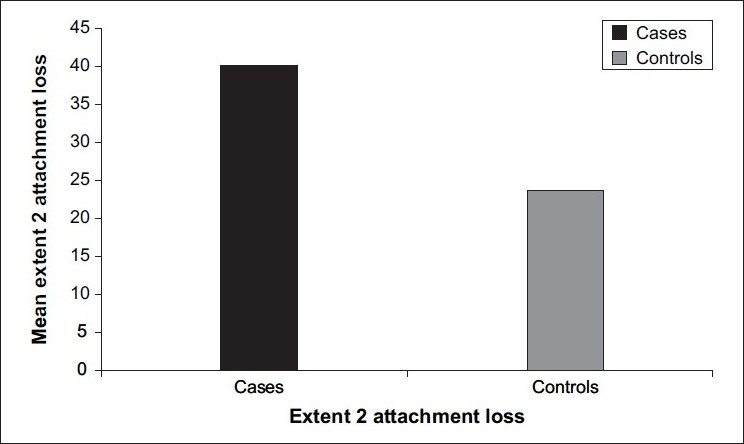
Mean value of Extent 2 attachment loss in cases and controls

Table 1 - Mean clinical attachment loss in cases (1.36±1.25) was significantly higher than that in controls (0.74±0.85) (*P*<0.0001) [Figure 1]. Mean bleeding index for cases (44.60±30.68) was higher than that for controls (37.01±27.49) (*P*=0.32), which is not statistically significant. Mean probing pocket depth in cases (3.12±1.02) was significantly higher than that in controls (2.83±1.00) (*P*=0.03). Mean simplified oral hygiene index for cases (2.95±1.22) was significantly higher than that for controls (2.35±1.13) (*P*<0.0001) [Figure 2]. Mean Extent 2 for cases (40.25±32.09) was significantly higher than that for controls (23.62±26.03) (*P*=0.001) [Figure 3]. Mean Extent 3 for cases (23.71±29.31) was significantly higher than that for controls (11.88±18.30) (*P*=0.004). Mean Extent 4 for cases (11.10±22.88) was significantly higher than that for controls (3.28±9.97) (*P*=0.02). Mean Severity 2 for cases (1.53±0.89) was significantly higher than that for controls (1.21±0.83) (*P*=0.007). Mean Severity 3 for cases (1.84±1.25) was significantly higher than that for controls (1.38±1.19) (*P*=0.006). Mean±standard deviation of Severity 4 for cases was 1.42±1.79; and for controls, it was 0.98±1.59 (*P*=0.07), which is not statistically significant. Mean±standard deviation of age for cases was 23.5±3.9; and for controls, it was 23.2±3.7 (*P*=0.55), which is not statistically significant. Mean±standard deviation of hemoglobin for cases was 9.36±1.49; and for controls, it was 9.82±1.29 (*P*=0.03), which is statistically significant.

**Table 1:** Mean±Standard deviation (SD) of different study variables in cases and controls

Variable	Cases	Controls	*P*-value*
		
	Mean±SD	Mean±SD	
Clinical attachment loss (CAL)	1.36±1.25	0.74±0.85	<0.0001# (Sig)
Bleeding Index (BI)	44.60±30.68	37.01±27.49	0.32# (NS)
Probing pocket depth (PPD)	3.12±1.02	2.83±1.00	0.03# (Sig)
Oral hygiene index (OHI)	2.95±1.22	2.35±1.13	<0.0001 (Sig)
Extent 2 (E2)	40.25±32.09	23.62±26.03	0.001# (Sig)
Extent 3 (E3)	23.71±29.31	11.88±18.30	0.004# (Sig)
Extent 4 (E4)	11.10±22.88	3.28±9.97	0.02# (Sig)
Severity 2 (S2)	1.53±0.89	1.21±0.83	0.007 (Sig)
Severity 3 (S3)	1.84±1.25	1.38±1.19	0.006 (Sig)
Severity 4 (S4)	1.42±1.79	0.98±1.59	0.07# (NS)
Age	23.5±3.9	23.2±3.7	0.55 (NS)
Hemoglobin	Hemoglobin	9.82±1.29	0.03 (Sig)

* P-values were calculated by using Student independent t test,

# Since variables were highly skewed, values were log transformed before using student independent t test.

Table 1, which gives the mean±standard deviation of different study variables and the test of significance of mean between cases and controls, indicates that cases had significantly more attachment loss, greater pocket depth, poor oral hygiene, more percentage of sites with clinical attachment loss ≥2, ≥3 and ≥4 mm (Extent 2, Extent 3 and Extent 4), more mean clinical attachment loss, i.e., ≥2, ≥3 per site (Severity 2 and Severity 3), and less hemoglobin than controls.

**Figure 4:**
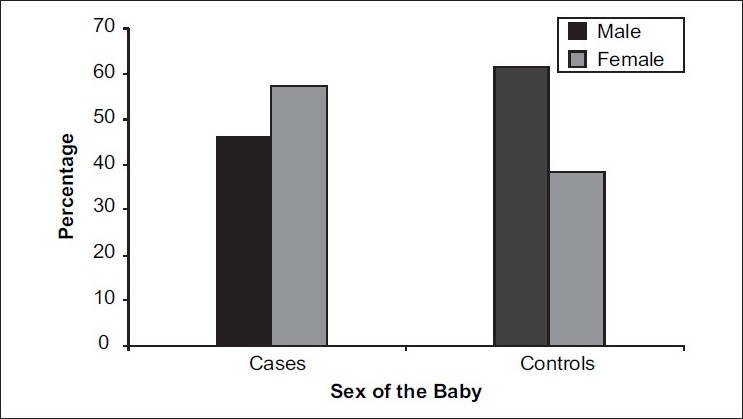
Sex ratio of the baby compared between cases and controls

**Figure 5:**
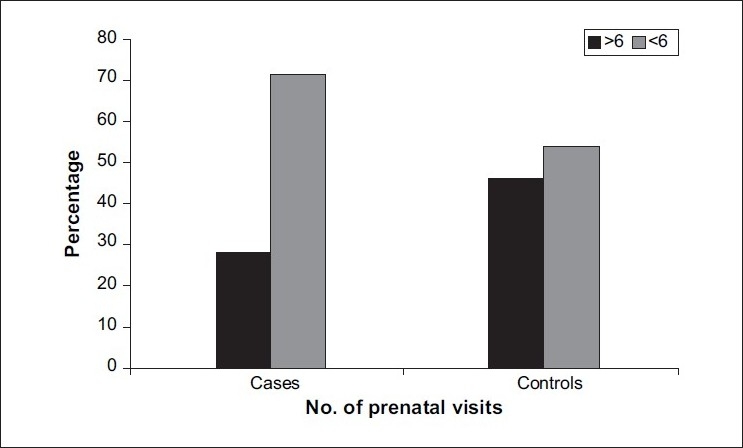
Proportion of prenatal visits between cases and controls

Table 2 gives the proportion of different characteristics in cases and controls. There is a significant difference in sex ratio between cases and controls. Proportion of female babies in cases (57.8%) was significantly higher than that of female babies in controls (38.5%) (*P*=0.04) [Figure 4]. There was a significant association between previous spontaneous abortions and increased incidence of preterm low birth weight in the current pregnancy The proportion of spontaneous abortions ≥1 in cases (20.2%) was significantly higher than that in controls (0%) (*P*<0.0001). There was a significant association between previous full term deliveries and decreased incidence of preterm low birth weight in the current pregnancy The proportion of previous full term=1, 2 and 3 (49%, 29.4%, 21.6%) was significantly higher in controls than in cases (22.1%, 14.7%, 3.7%) (*P*<0.0001). There was a significant difference in the number of prenatal visits between cases and controls. The proportion of number of prenatal visits <6 in cases (71.6%) was significantly higher than that in controls (53.9%) (*P*= 0.01); [Figure 5] while there was no significant association between cases and controls and other factors such as blood group, induced abortions, previous preterm deliveries, number of previous pregnancies, previous live births, Rh factor, hypertension, genitourinary tract infection, tobacco, alcohol and onset of prenatal care in contributing to an increased or decreased incidence of causing preterm low birth weight.

**Table 2:** Proportion of characteristics in cases and controls

Characteristics	Cases		Controls		Chi-square and *P*-value #
	No.	%	No.	%	
Sex of the baby					
Male	49	46.2	59	61.5	χ2=4.11
Female	57	57.8	37	38.5	*P*=0.04 (Sig)
Induced abortion					
0	106	97.3	98	96.1	*P*=0.71# (NS)
1	3	2.7	4	3.9	
Spontaneous abortion					
0	87	79.8	102	100	χ2=20.9
≥1	22	20.2	0	0	*P*<0.0001 (Sig)
Previous preterm					
0	104	95.4	102	100	*P*=0.06# (NS)
≥1	5	4.8	0	0	
Previous full term					
0	65	59.6	0	0	χ2=90.73
1	24	22.1	50	49	df=3
2	16	14.7	30	29.4	*P*<0.0001 (Sig)
3	4	3.7	22	21.6	
Number of previous pregnancies					
0	54	49.5	49	48.0	χ2=8.68
1	24	22.0	29	28.4	df=4
2	17	15.6	21	20.5	*P*=0.07 (NS)
3	10	9.8	3	2.94	
≥4	4	3.6	0	0	
Previous live births					
0	65	59.6	50	49	χ2=7.29
1	21	19.3	35	34.3	df=3
2	19	17.4	16	15.7	*P*=0.06 (NS)
3	4	3.7	1	0.98	
Blood group					
O	25	22.9	25	24.5	
A	20	18.3	18	17.6	χ2=2.30
B	26	23.9	21	20.6	df=5
A1	2	1.8	0	0.9	*P*=0.81 (NS)
AB	2	1.8	4	3.9	
A1B	1	0.9	0	0	
Rh factor					
Positive	72	94.7	66	95.7	*P*=1.00# (NS)
Negative	4	5.3	3	4.3	
Hypertension					
Yes	10	9.2	4	3.9	χ2=1.58
No	99	90.8	98	96.1	*P*=0.21 (NS)
Genitourinary tract infection					
Yes	12	11.0	12	11.8	χ2=0.002
No	97	89.0	90	88.2	*P*=0.96 (NS)
Tobacco/alcohol					
Yes	2	1.8	0	0	
No	107	98.2	102	100	*P*=0.50# (NS)
Onset of prenatal care					
Prior to 20 weeks	56	51.4	61	59.8	χ2=5.74
Between 20-25 weeks	47	43.1	31	30.4	df=3
After 25 weeks	3	2.8	8	7.8	*P*=0.12 (NS)
No care	3	2.8	2	2	
No. of prenatal visits					
≥6	31	28.4	47	46.1	χ2=6.30
<6	78	71.6	55	53.9	*P*=0.01 (Sig)

# P-values calculated from fisher exact test

Table 3 gives the results of multiple logistic regression analysis for cases and controls. It was found that the number of visits for prenatal care and percentage of sites with attachment loss ≥2 (Extent 2) were significant, and all other included variables were insignificant.

**Table 3:** Results of multiple logistic regression analysis – Cases vs. controls

Significant variables	Regression coefficient (b)	SE (b)	*P*-value	OR (95% CI)
No. of prenatal visits (<6)	0.81	0.38	0.03 (Sig)	2.25 (1.08-4.70)
Extent 2 (E2)	0.25	0.08	0.001 (Sig)	1.29 (1.10-1.51)

SE - Standard error; OR - Odds ratio; CI - Confidence interval

The variables number of visits for prenatal care and Extent 2 were found to be independently prognostic factors in cases. The odds ratio for number of prenatal visits was 2.25, which indicates that the risk of development of ‘cases’ in those whose number of prenatal visits is less than 6 compared to those with more than 6 prenatal visits is about twice. Similarly, the odds ratio for Extent 2 was 1.29, which indicates that for a 10-unit increment, Extent 2 contributes 29% more to the risk of development of ‘cases.’

## DISCUSSION

Recent studies indicate that periodontal infections contribute to the morbidity and mortality of individuals with certain systemic conditions, such as atherosclerosis, myocardial infarction, stroke and premature delivery.

Logistic regression techniques, which have been developed in the recent past, describe how periodontal infections independently contribute to the birth of a preterm baby even if other risk factors are present. Though periodontal infection may be only one factor among many, its relative importance does not diminish.[6]

The pathogenic mechanisms that have been hypothesized to cause preterm low birth weight as a consequence of periodontal infection are, translocation of bacteria or bacterial products / LPS to the fetoplacental unit to trigger the local release of biochemical mediators that elicit preterm delivery; or proinflammatory cytokines such as TNF-α, IL-1, IL-6 and PGE2 directly being translocated hematogenously to the fetoplacental unit.

Evidence to support this concept first came from experimental studies in pregnant hamster model, which demonstrated that experimental periodontitis as well as localized nondisseminating subcutaneous infections with Porphyromonas gingivalis could significantly retard fetal growth.[10,11]

Two preliminary human case-control studies conducted in the University of North Carolina provide further support to the hypothesis. In the first, mothers with periodontal disease were found to be at significantly greater risk for preterm low birth weight deliveries (7 times the odds) in comparison to periodontally healthy mothers. In the second study, levels of gingival crevicular fluid PGE_2_ as well as periodontal pathogens *Porphyromonas gingivalis, Tannerella forsythia (previously Bacteroides forsythus), Treponena denticola and Aggregatibacter actinomycetemcomitans (Previously Actinobacillus actinomycetemcomitans)* were found to be significantly elevated in mothers who underwent preterm low birth weight delivery as compared with control mothers, who had normal-term delivery.[4]

Low birth weight (LBW) is a major socioeconomic health problem worldwide.

Prematurity and LBW contribute to many medical and social problems for the child, such as neurodevelopmental problems, congenital anomalies, learning disabilities, etc.[12] From the standpoint of oral health, preterm and LBW are associated with poor calcification of teeth because of an inability to maintain proper calcium balance in the neonate. There is increased incidence of caries in LBW children, with significant hypoplasia of primary teeth. Palatal grooving caused by prolonged intubation is another orofacial problem. Tooth eruption may also be delayed into the mixed dentition.[13]

All of these findings indicate that periodontal disease must be viewed from a whole new perspective, particularly since some form of periodontal disease is present in a large percentage of the population.[14]

The present study was undertaken to identify if periodontitis contributed to preterm low birth weight.

The present study indicated that clinical attachment loss was significantly higher in preterm low birth weight cases. This is in accordance with the study conducted by Offenbacher *et al*. (1996),[8] who reported that preterm low birth weight cases had significantly worse periodontal disease than controls by attachment loss. In contrast, a study by Dempsey *et al*. (2000)[15] did not show statistical significance or association between CAL and preterm low birth weight.

In the present study, the extent scores E_2_, E_3_ and E_4_ and severity scores S_2_ and S_3_ were significantly higher in cases when compared to controls. Similarly in a study by Offenbacher *et al*. (1996),[8] Extent 3 scores demonstrated significant case-control differences. Severity scores were higher in cases but were not statistically significant.

Studies by Offenbacher *et al*. (1996)[8] and Mitchell-Lewis *et al*. (2000)[17] did not show an association between bleeding index and preterm low birth weight, a finding similar to that from the present study; but in contrast, in the studies done by Dempsey *et al*. (2000)[15] and Dasanayake (1998),[16] case mothers had significantly more bleeding sextants than controls.

The probing pocket depth was statistically significant in the present study. Cases tended to have higher probing pocket depth than controls. This is in accordance with the study by Davenport *et al*. (1998),[7] which showed that approximately half the study population had at least one deep pocket in at least one sextant in the mouth. In contrast, studies by Dempsey *et al*. (2000)[15] and Mitchell-Lewis *et al*. (2000)[17] did not show statistical significance for probing pocket depth. In a study by Offenbacher *et al*. (1996),[8] probing pocket depth was higher in cases than in controls, but it was not statistically significant.

In the present study, OHI-S score was statistically significant in comparison to a study by Dasanayake (1998),[16] which showed that case mothers had more sextants with calculus. Offenbacher *et al*. (1998)[12] have stated that the female gender could be a risk factor for low birth weight; in accordance, there was an association between female gender and preterm low birth weight in the present study. The proportion of female babies was more when compared to males. In contrast, a study by Dasanayake (1998)[16] showed no significant gender variation between case and control babies

The present study showed inadequate prenatal care as denoted by < 6 prenatal visits among cases to be statistically significant and to be associated with preterm low birth weight, which is in accordance with the studies by Dasanayake(1998),[16] Steven Gortmaker (1979)[18] and Jonathan Showstach (1984),[19] which showed inadequate prenatal care to increase the risk of low birth weight.

The present study showed a significant association between spontaneous abortion and preterm low birth weight. This is in accordance with the studies by Minkoff *et al*. (1984),[20] McGregor *et al*. (1990),[21] Elisabet Holst *et al*. (1994),[22] which showed that low birth weight was significantly correlated with a history of previous abortions.

The present study did not show any association between the number of previous pregnancies and preterm low birth weight, which is in contrast to the study by Minkoff *et al*. (1984).[20]

Studies by Hillier *et al*. (1995)[23] and Minkoff *et al*. (1984)[20] showed that a history of prior preterm birth was associated with a risk of preterm labor and preterm birth in the current pregnancy. In contrast, the present study did not show an association of recognized risk factors such as previous preterm delivery.

The present study did not find an association between parity (live births) and preterm low birth weight, which is in accordance with the study by Offenbacher *et al*. (1996),[8] where no significant difference in parity was found between cases and controls.

Williams CECS *et al*. (2000)[2] have stated that smoking and other forms of tobacco use and alcohol use were associated with preterm low birth weight. This was also found in the studies by Butler (1972)[24] and Halliday (1982).[25] In contrast, the present study did not show an association between tobacco or alcohol use and preterm low birth weight similar to a study by Offenbacher *et al*. (1996),[8] where alcohol consumption and smoking were not associated with preterm low birth weight.

Studies by Meis *et al*. (1995),[26] Minkoff *et al*. (1984),[20] Mfller *et al*. (1984),[27] Krohn *et al*. (1991),[28] Holst *et al*. (1993)[22] and Hillier *et al*. (1995)[23] have shown an association between preterm low birth weight and genitourinary tract infection; in contrast, the present study did not show any correlation between genitourinary tract infection and preterm low birth weight.

The present study showed an association between previous full-term deliveries and preterm low birth weight; cases had significantly less number of previous full-term deliveries than controls. Similarly there was an association between low hemoglobin levels (anemia) and preterm low birth weight; but there was no association between onset of prenatal care, blood group, Rh factor, hypertension and preterm low birth weight.

Inadequate prenatal care was found to be an independent prognostic factor in cases in the present study; but in contrast, the study by Offenbacher *et al*. (1996)[8] did not show inadequate prenatal care to be a risk factor.

Results of multivariate logistic regression analysis in a study by Offenbacher *et al*. (1996),^8^] showed that the Extent 3 variable, which was chosen as a periodontal disease predictor variable, was found to be significantly associated with preterm low birth weight. In comparison, in the present study the Extent 2 variable was an independent prognostic factor of preterm low birth weight.

Numerous studies — by Damare *et al*. (1995),[29] Offenbacher *et al*. (1996,[8] 1998),[6,12] Davenport *et al*. (1998),[7] Lieff *et al*. (2000),[30] Jeffcoat *et al*. (2001)[31] — have shown an association between periodontal disease and preterm low birth weight and indicated that periodontal disease could be an independent risk factor for preterm low birth weight. The present study, in accordance with these and various other studies, showed that periodontal disease could be a risk factor for preterm low birth weight.

## CONCLUSION

In the present study, the number of visits for prenatal care and Extent 2 remained significant when compared to other variables; thus the lesser the number of visits for prenatal care and the more the extent of attachment loss (percentage of sites with CAL ≥2 mm), the higher is the risk for delivering a preterm low birth weight infant.

Therefore, the present study indicates that periodontal infection is a contributing factor for preterm low birth weight. However, more prospective studies are needed to confirm whether periodontal infection could act as an independent risk factor for preterm low birth weight.

## References

[1] Hill GB (1998). Preterm birth: associations with genital and possibly oral microflora. Ann Periodontol.

[2] Williams CECS, Davenport ES, Sterne JAC, Sivapathasundaram V, Fearne JM, Curtis MA (2000). Mechanisms of risk in preterm low birth weight infants. Periodontol 2000.

[3] Mealey BL (1999). Influence of periodontal infections on systemic health. Periodontol.

[4] Champagne CM, Madianos PN, Lieff S, Murtha AP, Beck JD, Offenbacher S (2000). Periodontal medicine: emerging concepts in pregnancy outcomes. J Int Acad Periodontol.

[5] Page RC (1998). The pathobiology of periodontal diseases may affect systemic diseases: inversion of a paradigm. Ann Periodontol.

[6] Offenbacher S, Beck JD, Lieff S, Slade G (1998). Role of periodontitis in systemic health: spontaneous preterm birth. J Dent Educ.

[7] Davenport ES, Williams CE, Sterne JA, Sivapathasundram V, Fearne JM, Curtis MA (1998). The East London Study of Maternal Chronic Periodontal Disease and Preterm Low Birth Weight Infants: study design and prevalence data. Ann Periodontol.

[8] Offenbacher S, Katz V, Fertik G, Collins J, Boyd D, Maynor G (1996). Periodontal infection as a possible risk factor for preterm low birth weight. J Periodontol.

[9] Carlos JP, Wolfe MD, Kingman A (1986). The extent and severity index: a simple method for use in epidemiologic studies of periodontal disease. J Clin Periodontol.

[10] Collins JG, Windley HW, Arnold RR, Offenbacher S (1994). Effects of a Porphyromonas gingivalis infection on inflammatory mediator response and pregnancy outcome in hamsters. Infect Immun.

[11] Collins JG, Kirtland BC, Arnold RR, Offenbacher S (1995). Experimental Periodontitis retards hamster foetal growth. J Dent Res.

[12] Offenbacher S, Jared HL, O’Reilly PG, Wells SR, Salvi GE, Lawrence HP (1998). Potential pathogenic mechanisms of periodontitis associated pregnancy complications. Ann Periodontol.

[13] Casamassimo PS (2001). Maternal oral health. Dent Clin North Am.

[14] Drisko CH (2000). Trends in surgical and non surgical periodontal treatment. JADA.

[15] Dempsey R, Bissada N, Ashmead G, Clapp J, Amini S (2000). Is periodontitis a risk factor for preterm labour and/or low birth weight?S. J Dent Res.

[16] Dasanayake AP (1998). Poor periodontal health of the pregnant woman as a risk factor for low birth weight. Ann Periodontol.

[17] Mitchell Lewis DA, Papapanou PN, Engebretson S, Grbic J, Herrera Abreu M, Celenti R (2000). Periodontal intervention decreases the risk of preterm low birth weight. J Dent Res.

[18] Gortmaker SL (1979). The effects of prenatal care upon the health of the newborn. Am J Public Health.

[19] Showstack JA, Budetti PP, Minkler D (1984). Factors associated with birthweight: an exploration of the roles of prenatal care and length of gestation. Am J Public Health.

[20] Minkoff H, Grunebaum AN, Schwarz RH, Feldman J, Cummings M, Crombleholme W (1984). Risk factors for prematurity and premature rupture of membranes: a prospective study of the vaginal flora in pregnancy. Am J Obstet Gynecol.

[21] McGregor JA, French JI, Richter R, Franco-Buff A, Johnson A, Hillier S (1990). Antenatal microbiologic and maternal risk factors associated with prematurity. Am J Obstet Gynecol.

[22] Holst E, Goffeng AR, Andersch B (1994). Bacterial vaginosis and vaginal microorganisms in idiopathic premature labor and association with pregnancy outcome. J Clin Microbiol.

[23] Hillier SL, Nugent RP, Eschenbach DA, Krohn MA, Gibbs RS, Martin DH (1995). Association between bacterial vaginosis and preterm delivery of a low-birth-weight infant. The Vaginal Infections and Prematurity Study Group. N Engl J Med.

[24] Butler NR, Goldstein H, Ross EM (1972). Cigarette smoking in pregnancy: its influence on birth weight and perinatal mortality. Br Med J.

[25] Halliday HL, Reid MM, McClure G (1982). Results of heavy drinking in pregnancy. Br J Obstet Gynaecol.

[26] Meis PJ, Goldenberg RL, Mercer B, Moawad A, Das A, McNellis D (1995). The preterm prediction study: significance of vaginal infections. National Institute of Child Health and Human Development Maternal-Fetal Medicine Units Network. Am J Obstet Gynecol.

[27] Møller M, Thomsen AC, Borch K, Dinesen K, Zdravkovic M (1984). Rupture of fetal membranes and premature delivery associated with group B streptococci in urine of pregnant women. Lancet.

[28] Krohn MA, Hillier SL, Lee ML, Rabe LK, Eschenbach DA (1991). Vaginal *Bacteroides* species are associated with an increased rate of preterm delivery among women in preterm labor. J Infect Dis.

[29] Damare SM, Maynor G, Jenzano J, Katz V, Offenbacher S (1995). Relationship between periodontal and amniotic fluid inflammatory mediators in pregnancy. J Dent Res.

[30] Lieff S, Jared H, McKaig R, Herbert W, Murtha A, Moss K (2000). Periodontitis and preterm low birth weight risk in pregnant women. J Dent Res.

[31] Jeffcoat MK, Geurs NC, Reddy MS, Cliver SP, Goldenberg RL, Hauth JC (2001). Periodontal infection and preterm birth: results of a prospective study. J Am Dent Assoc.

